# Clinical Reports

**Published:** 1901-03

**Authors:** S. J. J. Harger

**Affiliations:** Veterinary Department, University of Pennsylvania


### CLINICAL REPORTS.

By S. J. J. Harger, V.M.D.,
VETERINARY DEPARTMENT, UNIVERSITY OF PENNSYLVANIA.

Osteoma in the Lungs. Osteomas are clinically separated into two groups: (1) Those developing upon bone tissue; (2) those affecting tissues other than bone. The former include the exostoses and enostoses (found in the medullary canal). The latter, exceedingly rare, have been seen in serous membranes and the walls of bloodvessels.

Concerning the present case, the animal, a gelding, was a dissecting subject of which no ante-mortem examination was made. The posterior lobe of the left lung was found to be the seat of pneumonia over a large area, which presented a number of smaller and larger abscesses. In the interior of the hepatized area a hard tumor the size of a child's head was found. When sawed into the section presented the appearance of cancellated bone tissue, somewhat more compact than that seen in the epiphyses of long bones. Here and there were seen thin bridles of lung tissue. Microscopical examination showed its composition to be that of true bone—an osteoma. Of its mode of formation I can only conjecture; probably it was secondary, resulting from the conversion into bone of the exudate in the inflamed lung. Clinically it might have been mistaken for a perfectly solidified pneumonic area. I have never seen osteoma in this organ recorded in the literature. The prognosis in such a case would be as unsatisfactory as the diagnosis.

Treatment of Azoturia. Veterinary practitioners employ different medicinal formulas in the treatment of the same disease. The favorite prescription contains more virtue than any other treatment, although every practitioner claims the same degree of success—an apparent incongruity which I shall not endeavor to harmonize here. Every veterinarian has his peculiar treatment for azoturia, and the one outlined here is mentioned simply because it has apparently shown some advantages over others.

*Treatment.* Phlebotomy of the jugular vein, five to seven quarts of blood being abstracted, according to the condition of plethora of the animal. Eight ounces of sodium bicarbonate and sodium sulphate are administered in a drench and hot blankets applied to the loins for the first day or two. This treatment should be supplemented by hypodermatic injections of sulphate of strychnine, $\frac{1}{2}$ to 1 grain three times daily, according to the size and temperament of the horse, until the animal is able to get up and walk ; the catheter is also used. No diuretics are employed. The therapeutic analysis of the treatment will not be dwelt upon here.

Forty-four cases were treated in the above manner. Of these sixteen (36 per cent.) recovered. One may say that this is a small percentage of recoveries, but one must discriminate between the conditions met in general practice and those seen in hospital work. Almost invariably the subjects were animals of the coarsest, most lymphatic, and heavy draught type, highly fed and plethoric —animals in which the disease is aggravated, has a rapid course, and tends to a rapid, fatal termination. Again, most frequently a hospital is sought for only as a last resort.

Taking everything into consideration, the above treatment seems to have been satisfactory. According to Lucet (*Revue Vétér.*), the percentage of recoveries varies from 20 to 40 per cent.

Colic ; Hernia of Stomach through Ruptured Mesentery. Gray gelding, aged. History : Examined Wednesday morning ; colicky pains since Tuesday morning.

*Symptoms.* Abdominal pain, pawing, recumbent position, and tendency to balance himself on his back ; temperature slightly elevated; pulse and respirations accelerated; defecation seen once on previous day. Died during the night.

*Post-mortem.* This revealed a new lesion, causing symptoms included under the vague term " colic." The omentum of the small intestine toward its anterior portion showed a long, vertical rent and torn away for several inches from the smaller curvature of the bowel. The stomach was rotated on an antero-posterior axis toward the left, the left extremity being pushed through the hole in the mesentery. The small intestine, for about two and a half feet corresponding to this point and encircling the stomach around the two curvatures, was coal-black, gangrenous, and the mucous membrane ulcerated. The circulation was completely arrested and the blood coagulated *in situ* from obstruction to the venous circulation. The treatment consisted of eserine and pilocarpine, followed, when there was no relief by narcotics, and mustard to the abdomen.

The impossibility of making a diagnosis and the inefficiency of the treatment are very evident.

